# Use of a geographic information system to track smelter-related lead exposures in children: North Lake Macquarie, Australia, 1991–2002

**DOI:** 10.1186/1476-072X-5-30

**Published:** 2006-07-19

**Authors:** Alan Willmore, Tim Sladden, Lucy Bates, Craig B Dalton

**Affiliations:** 1 Centre for Epidemiology and Research, NSW Department of Health, Sydney, NSW, Australia; 2 HIV/AIDS & STI Surveillance, Public Health Programme, Secretariat of the Pacific Community Noumea, New Caledonia, Australia; 3 Hunter New England Population Health, Hunter New England Area Health Service, NSW, Australia; 4 School of Medical Practice and Population Health, University of Newcastle, NSW, Australia

## Abstract

**Background:**

To determine patterns of childhood lead exposure in a community living near a lead and zinc smelter in North Lake Macquarie, Australia between 1991 and 2002.

**Methods:**

An analysis of serial blood lead levels (BLL) of children less than 13 years of age in North Lake Macquarie participating in voluntary blood lead screening. Distance to the smelter and soil lead concentration of the child's place of residence was calculated. Categorical analysis of BLL by residential distance from smelter, residential soil lead concentration, age and year of sample was calculated. Linear regression models were fit for blood lead levels against residential distance from smelter, the log of residential soil lead concentration, age and year of BLL sample.

**Results:**

Geometric mean BLLs were statistically significantly higher for distances less than 1.5 kilometres from the smelter and for residential soil lead concentrations greater than 300 ppm. Yearly BLLs since 1995 were statistically significantly lower than for preceding years, with an average decrease of 0.575 μg/dL per year since 1991. BLLs are statistically significantly higher for children whose age is 1 to 3 years old. Linear regression modelling of BLL predicted a statistically significant decrease in BLL of 3.0831 μg/dL per kilometre from the smelter and a statistically significant increase in BLL of 0.25 μg/dL per log of lead in residential soil. The model explained 28.2% of the variation in BLL.

**Conclusion:**

Residential distance to the smelter, log of residential soil lead concentration, child's age and year of BLL sample are statistically significant factors for predicting elevated BLLs in children living near a North Lake Macquarie lead smelter.

## Background

**Figure 1:**
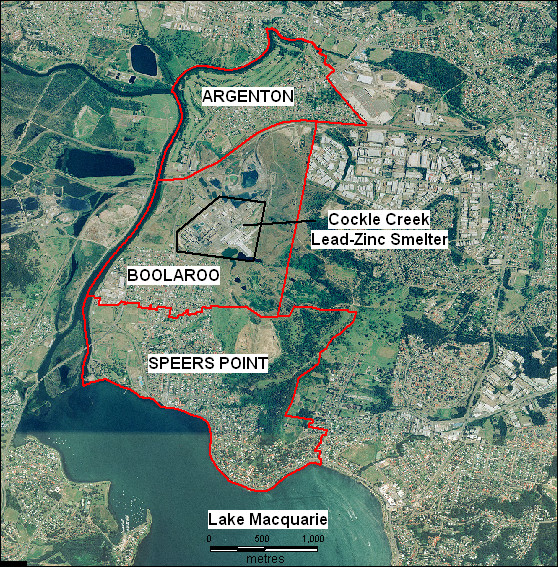
Location of the Cockle Creek lead-zinc smelter in relation to the suburbs Boolaroo, Argenton and Spears Point, North Lake Macquarie, NSW, Australia.

Newcastle is a city on the Australian eastern seaboard with a history of heavy industry including coal mining; metal smelting, production and fabrication; and mineral export via a deep water port facility. Over time, heavy industry in the area has been scaled back, including the recent closure of the Cockle Creek lead-zinc smelter in September 2003. The smelter is situated approximately 20 kilometres southwest of Newcastle city on Cockle Creek, which drains into the northern end of Lake Macquarie. Three residential suburbs lie adjacent to the smelter site – Argenton to the north, Boolaroo immediately south and Speers Point to the southeast (see Figure 1).

The smelter originally commenced operation in 1897, with zinc and lead production until 1922. Production resumed in 1962 subsequent to significant residential development around the site.

The sources of lead exposure to the community include the smelter stack emissions and fugitive emissions resulting directly from various aspects of the smelting process. Other lead exposure has resulted from use of smelter slag as landfill in numerous sites (both private and public) across Lake Macquarie council area. Recent research suggests the lead in this slag may be bioaccessible, making it available for absorption into the blood stream during human biological processes, such as digestion [1].

Hunter Area Health Service commenced investigations into child resident blood lead levels (BLLs) in the North Lake Macquarie (NLM) area during May 1991 in response to a local parliamentary task force request. The study area included the two suburbs most directly affected by the smelter; Argenton and Boolaroo [2].

Pilot soil surveys revealed lead contamination of the area surrounding the smelter [3], leading to an extensive soil survey in 1992. An Environmental Health Centre (EHC) was established in Boolaroo to work specifically on issues of lead exposure in the community. Free child BLL testing using the venous method commenced initially in Boolaroo and later, due to community concern, extended to child residents of Argenton and Speers Point. Child blood lead testing has been offered annually to all child residents aged less than 13 years, and more frequently where an individual's BLL has been found to be above the national goal of 10 micrograms per decilitre (μg/dL) of blood as defined by the Australian National Health and Medical Research Council (NHMRC) [4].

Numerous studies have described the relationship between distance of residence from smelter and elevated soil lead levels being important indicators for elevated blood lead levels in children [5] – [11]. This paper re-analyses the soil lead survey data and the ongoing BLL testing of child residents in the three suburbs adjacent to the smelter to illustrate changing patterns of lead exposure in the affected community between 1991 and 2002. Both residential soil lead concentration from the 1992 soil lead survey and individual child lead levels were mapped and analysed with a Geographic Information System (GIS), and linear regression models of BLLs were fit with the explanatory variables; residential distance from the smelter, 1992 residential soil lead concentration, year of BLL sample and age of child.

## Results

**Figure 2:**
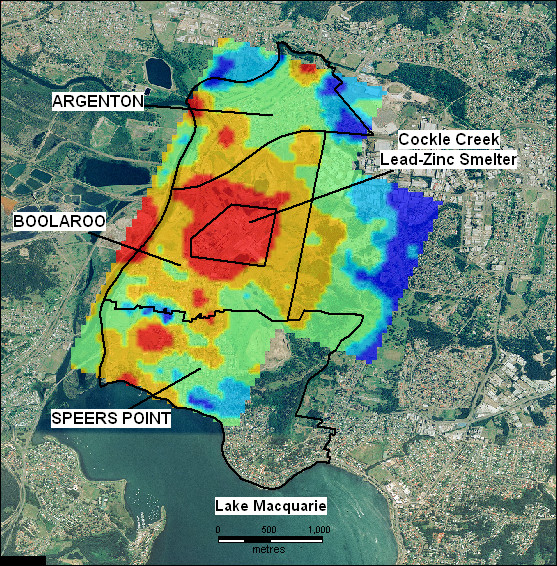
Gridded and imaged 1992 soil lead concentration in the suburbs surrounding the Cockle Creek lead-zinc smelter, North Lake Macquarie, NSW, Australia. Navy: soil lead < 150 ppm, Turquoise: 150 ppm – < 300 ppm, Green: 300 ppm – < 1000 ppm, Orange: 1000 ppm – <4000 ppm, Red: ≥ 4000 ppm

Gridded and imaged 1992 soil lead data is presented in Figure 2, with orange and red areas highlighting where soil lead levels are greater than 1000 ppm, which was the NHMRC level acceptable for the living environment at the time of a pilot soil survey in 1991. The area immediately around the smelter has the highest soil lead values; however there are isolated 'hot spots' within the community surrounding the smelter where values exceed 1000 ppm lead.

**Figure 3:**
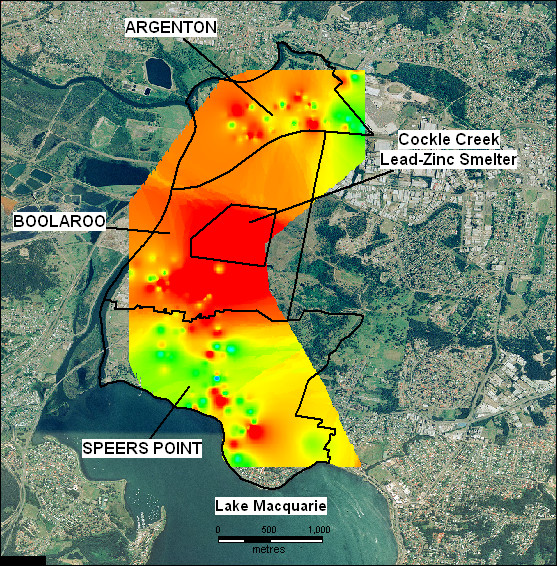
Gridded and imaged 1992 blood lead levels of child residents in the suburbs surrounding the Cockle Creek lead-zinc smelter, North Lake Macquarie, NSW, Australia. Navy: BLL < 5.5 μg/dL, Turquoise: 5.5 μg/dL – < 7.5 μg/dL, Green: 7.5 μg/dL – < 10 μg/dL, Yellow: 10 μg/dL (the NHMRC national level of concern) – < 15 μg/dL, Red: ≥ 15 μg/dL.

**Figure 4:**
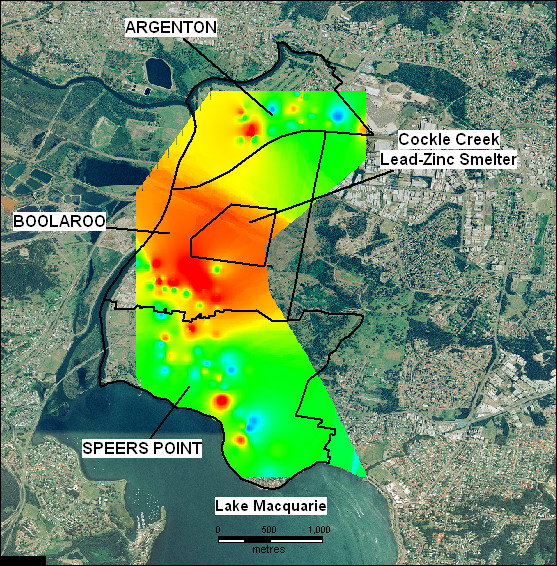
Gridded and imaged 1996 blood lead levels of child residents in the suburbs surrounding the Cockle Creek lead-zinc smelter, North Lake Macquarie, NSW, Australia. Navy: BLL < 5.5 μg/dL, Turquoise: 5.5 μg/dL – < 7.5 μg/dL, Green: 7.5 μg/dL – < 10 μg/dL, Yellow: 10 μg/dL (the NHMRC national level of concern)- < 15 μg/dL, Red: ≥ 15 μg/dL.

**Figure 5:**
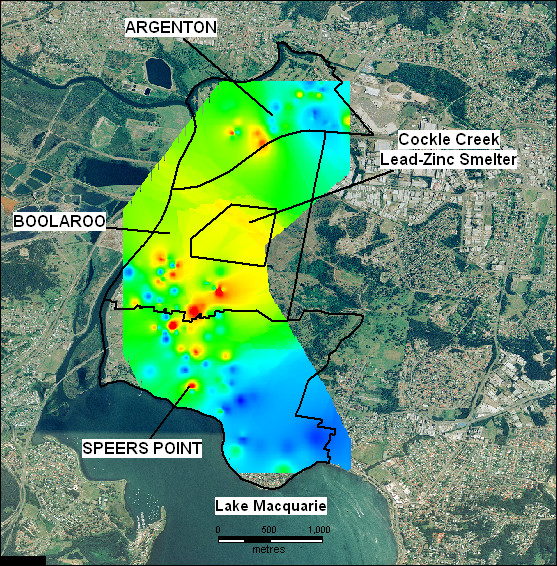
Gridded and imaged 2002 blood lead levels of child residents in the suburbs surrounding the Cockle Creek lead-zinc smelter, North Lake Macquarie, NSW, Australia. Navy: BLL < 5.5 μg/dL, Turquoise: 5.5 μg/dL – < 7.5 μg/dL, Green: 7.5 μg/dL – < 10 μg/dL, Yellow: 10 μg/dL (the NHMRC national level of concern)- < 15 μg/dL, Red: ≥ 15 μg/dL.

Table 1 shows the geometric mean BLLs of children by year. Table 2 shows the geometric mean BLLs for the indicators: distance from smelter, soil lead concentration of residence and child's age. Geometric mean BLLs across all years and indicators is 8.31 μg/dL (95% C.I. 8.14 – 8.48). Figures 3, 4, 5 show gridded and imaged BLL data for years 1992, 1996 and 2002 respectively and highlight the decreasing BLL values since 1991 as well as the decrease in BLL with increasing distance from the smelter. Figure 3 shows that child BLL concentrations at the start of the study period (1992) are very high compared to the levels at the end of the study period (2002) in Figure 5. Figures 4 and 5 show a distinct gradient of exposure with children residing closest to the smelter experiencing the highest BLL levels (geometric mean 10.83 μg/dL for all children sampled 1991 – 2002 living less than 1 kilometre from the smelter). As distance from the smelter increases the BLLs decrease (geometric mean 6.24 μg/dL for all children sampled 1991 – 2002 living more than 2.5 kilometres from the smelter). The images of BLL concentration are not uniform with some individuals close to the smelter having relatively low levels and other children more distant to the smelter experiencing high BLLs. Despite this, there is a clear overall trend of decreasing BLL with distance from source and this is repeated in each annual BLL image (not all years shown).

**Table 1:** Annual blood lead levels (μg/dL) in NLM children 0–12 years.

**Year**	**N**	**Min, max (range)**	**Geometric mean ( ± 95% CIs)**	**Median (25**^th^**, 75**^th^**percentiles)**
1991	215	4, 38 (34)	12.82 (12.15 – 13.54)	13 (10,17)
1992	270	2, 39 (37)	10.73 (10.17 – 11.33)	11 (8, 14)
1993	326	1, 44 (43)	10.14 (9.66 – 10.64)	10 (8, 14)
1994	251	3, 34 (31)	9.80 (9.31 – 10.33)	10 (7, 13)
1995	243	2, 30 (28)	8.49 (8.01 – 9.00)	8 (6, 11)
1996	181	3, 34 (31)	8.63 (8.05 – 9.25)	8 (6, 12)
1997	208	2, 21 (19)	7.18 (6.72 – 7.67)	7 (5, 10)
1998	213	2, 25 (23)	6.97 (6.53 – 7.45)	7 (5, 10)
1999	170	2, 37 (35)	7.90 (7.36 – 8.49)	8 (6, 11)
2000	209	2, 24 (22)	6.72 (6.30 – 7.17)	6 (5, 9)
2001	287	1, 24 (23)	5.76 (5.39 – 6.16)	6 (4, 9)
2002	193	1, 36 (35)	6.26 (5.69 – 6.87)	6 (4, 10)
Total	2766	1, 44 (43)	8.31 (8.14 – 8.48)	8 (6, 12)

**Table 2:** BLLs (μg/dL) in NLM children 0–12 years by residential distance from smelter, soil lead concentration (1992) and age group.

**Indicator**	**N**	**Min, max** **(range)**	**Geometric mean** **( ± 95% CIs)**	**Median** **(25**^**th**^**, 75**^**th**^**percentiles)**
**Distance**				
0 – < 1 km	558	1, 38 (37)	10.83 (10.36 – 11.32)	11 (8, 16)
1 – < 1.5 km	980	1, 36 (35)	8.76 (8.49 – 9.04)	9 (6, 12)
1.5 – < 2 km	812	2, 35 (33)	7.28 (7.05 – 7.52)	7 (5, 10)
2 – < 2.5 km	275	1, 44 (43)	6.90 (6.41 – 7.43)	7 (5, 10)
≥ 2.5 km	141	1, 26 (25)	6.24 (5.67 – 6.86)	6 (5, 9)
				
**Soil lead (1992 soil survey)**				
Resident outside soil survey area	201	1, 38 (37)	6.08 (5.61 – 6.59)	6 (4, 9)
0 – < 150 ppm	201	2, 35 (33)	7.05 (6.58 – 7.54)	7 (5, 9)
150 – < 300 ppm	278	1, 44 (43)	7.19 (6.72 – 7.70)	7 (5, 10)
300 – < 1000 ppm	1085	1, 30 (29)	8.04 (7.81 – 8.28)	8 (6, 11)
1000 – < 2000 ppm	537	2, 36 (34)	9.37 (8.98 – 9.77)	10 (7, 13)
≥ 2000 ppm	464	1, 38 (37)	10.48 (9.95 – 11.03)	11 (8, 15)
				
Age Group				
Less than 1 yr	58	2, 34 (32)	7.54 (6.50 – 8.75)	7 (5, 10)
1 yr	154	1, 38 (37)	9.98 (9.10 – 10.94)	10 (8, 14)
2 yrs	175	2, 44 (42)	10.81 (9.92 – 11.78)	11 (7, 17)
3 yrs	177	2, 38 (36)	10.11 (9.27 – 11.02)	10 (7, 15)
4 yrs	208	1, 26 (25	8.79 (8.14 – 9.49)	9 (6, 13)
5 yrs	297	1, 38 (37)	8.98 (8.43 – 9.56)	9 (6, 13)
6 yrs	301	2, 29 (27)	8.43 (7.97 – 8.91)	9 (6, 12)
7 yrs	283	2, 26 (24)	8.38 (7.95 – 8.85)	8 (6, 12)
8 yrs	291	1, 30 (29)	7.88 (7.43 – 8.37)	8 (6, 11)
9 yrs	269	1, 26 (25)	7.57 (7.11 – 8.07)	8 (5, 11)
10 yrs	237	1, 24 (23)	7.06 (6.62 – 7.52)	7 (5, 10)
11 yrs	225	1, 23 (22)	6.77 (6.33 – 7.24)	7 (5, 10)
12 yrs	91	2, 18 (16)	6.72 (6.16 – 7.34)	7 (5, 9)
Total	2766	1, 44 (43)	8.31 (8.14 – 8.48)	8 (6, 12)

**Figure 6:**
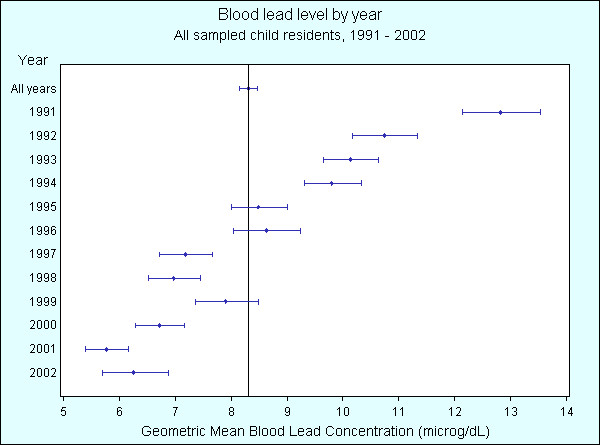
Geometric mean blood lead levels (with 95% confidence interval) of child residents in the suburbs surrounding the Cockle Creek lead-zinc smelter by year, North Lake Macquarie, NSW, Australia, 1991 – 2002.

**Figure 7:**
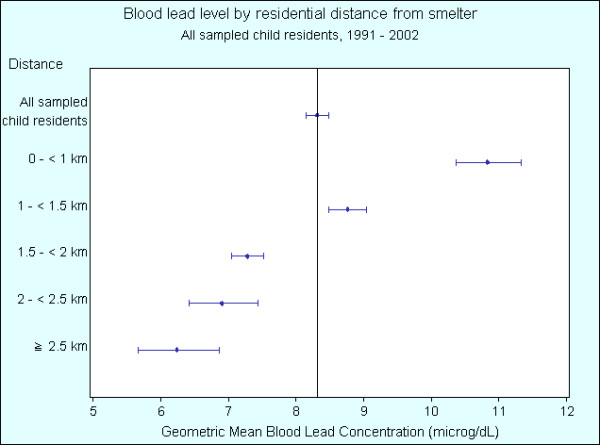
Geometric mean blood lead levels (with 95% confidence interval) of child residents by distance from the Cockle Creek lead-zinc smelter, North Lake Macquarie, NSW, Australia, 1991 – 2002.

**Figure 8:**
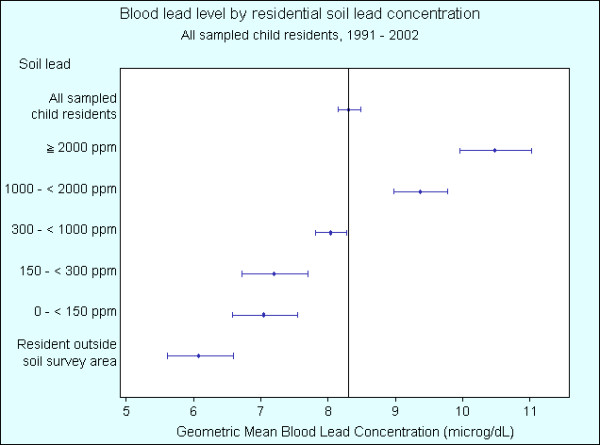
Geometric mean blood lead levels (with 95% confidence interval) of child residents in the suburbs surrounding the Cockle Creek lead-zinc smelter by 1992 residential soil lead concentration, North Lake Macquarie, NSW, Australia, 1991 – 2002.

**Figure 9:**
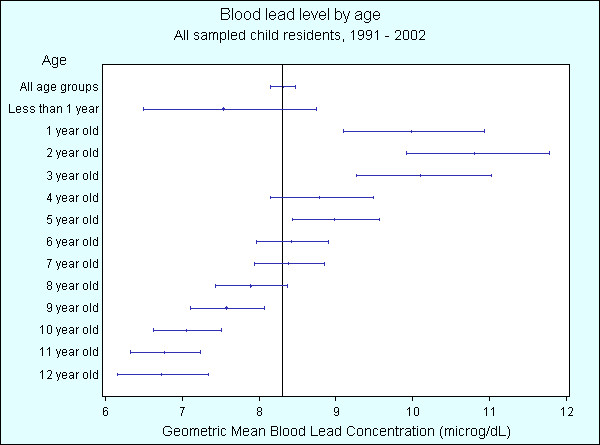
Geometric mean blood lead levels (with 95% confidence interval) of child residents in the suburbs surrounding the Cockle Creek lead-zinc smelter by age, North Lake Macquarie, NSW, Australia, 1991 – 2002.

Compared to the geometric mean BLL for all children, Figure 6 shows that geometric mean BLLs are statistically significantly higher for the years 1991 – 1994, and statistically significantly lower for years 1997, 1998, and 2000 – 2002. Figure 7 shows that geometric mean BLLs are statistically significantly higher for children living less than 1.5 km from the smelter, and statistically significantly lower for children living greater than 1.5 km. Figure 8 shows that geometric mean BLLs are statistically significantly higher for children whose 1992 residential soil lead concentration is greater than 1000 ppm, and statistically significantly lower for children whose 1992 residential soil lead concentration is less than 1000 ppm. Figure 9 shows that geometric mean BLLs are statistically significantly higher for children whose age is 1 to 3 years old, and statistically significantly lower for children whose age is 9 to 12 years old.

Linear modelling of the dependent variable BLL was undertaken with the explanatory variables year, age and residential distance from smelter. A non-linear relationship between BLL and residential soil lead was observed so the log of residential soil lead concentration was used in the model. It was also observed in Figure 9 that there was a non-linear relationship between BLL and age, with BLL increasing from birth until 3 years of age where it then decreases. Therefore higher order polynomial terms of age (quadratic and cubic) were included in the model. The resulting model is:

BLL = 15.913 - 3.0831 * Dist + 0.2503 * log Soil - 0.5747 * Year

    + 1.01101 * Age - 0.21409 * Age^2 ^+ 0.0091 * Age^3^

where Dist = residential distance (km) from smelter

log Soil = the natural log of residential soil lead concentration (ppm)

Age = age when BLL sample taken

Year = year when BLL sample taken from 1991

Age^2 ^= age squared

Age^3 ^= age cubed

The SAS v8.2 [12] output for the model is presented in Table 3. The model explains 28.2% of the variation in BLL with all parameter estimates statistically significant (95% confidence).

**Table 3:** SAS output from linear modelling of BLLs in children by residential distance from smelter and soil lead concentration, year and age.

**The REG Procedure** **Dependent Variable: BLL Blood Lead**						
						
**Analysis of Variance**						
	
**Source**	**DF**	**Sum of squares**	**Mean square**	**F value**	**Pr > F**	
	
Model	6	20174	3362.35333	165.59	<.0001	
Error	2532	51414	20.30563			
Corrected total	2538	71588				
	
						
			
**Root MSE**	4.50618	**R-Square**	0.2818			
**Dependent Mean**	9.75935	**Adj R-Sq**	0.2801			
**Coeff Var**	46.17290					
			
						
**Parameter Estimates**						
**Variable**	**Label**	**DF**	**Parameter estimate**	**Standard error**	**t value**	**Pr > |t|**

Intercept	Intercept	1	15.91313	1.24889	12.74	<.0001
dist	Distance from smelter (km)	1	-3.08310	0.29730	-10.37	<.0001
yr_norm	Normalised year	1	-0.57469	0.02556	-22.49	<.0001
age	Age	1	1.01101	0.34306	2.95	0.0032
agesq	Age squared	1	-0.21409	0.05774	-3.71	0.0002
agecb	Age cubed	1	0.00910	0.00286	3.18	0.0015
soil_pb_ln	Log of residential soil lead (ppm)	1	0.25032	0.11501	2.18	0.0296

**Parameter Estimates**						
		
**Variable**	**Label**	**Standardized estimate**	**Lower 95% confidence limit**	**Upper 95% confidence limit**		
		
Intercept	Intercept	0	13.46419	18.36208		
dist	Distance from smelter (km)	-0.23354	-3.66607	-2.50013		
yr_norm	Normalised year	-0.38032	-0.62480	-0.52457		
age	Age	0.60008	0.33830	1.68372		
agesq	Age squared	-1.74455	-0.32731	-0.10088		
agecb	Age cubed	0.90323	0.00349	0.01471		
soil_pb_ln	Log of residential soil lead (ppm)	0.04903	0.02481	0.47584		

## Discussion

This paper summarises results of a soil lead survey, and of ongoing blood lead monitoring of children who have been exposed to a variety of lead sources emanating from a lead-zinc smelter near North Lake Macquarie in New South Wales, Australia. Results have been mapped geographically to indicate exposure gradients away from the smelter point source over time. The results show that child blood lead levels in the NLM area are higher for those living closer to the smelter, are higher where the 1992 residential soil lead concentration is higher and higher for the very young (ages 1–3). However, BLLs have been decreasing over time due to reduced emissions from the smelter, lead abatement activities and education programs leading to a reduction in behaviour-related exposure.

Blood lead monitoring continues in NLM child residents. Since monitoring started in 1991, various factors have influenced the response rate of local residents to having their children tested for lead exposure. Intensive recruitment drives have occurred at various times (1993, 1994 and from 2000 onwards) and testing offered at the three local primary schools since 1992 and monthly at the EHC since 1999. However, lead testing is voluntary and significant numbers of families elect not to have their children tested for a number of reasons. Therefore the children who are tested each year may not be representative of all the children in the area. Community attitudes to the blood lead testing program vary and a number of barriers to testing have been identified [10]. Repeat testing has also focussed on those children having elevated BLLs. All these factors influence how representative the children tested are of the broader target population (all children under 14 years residing in the NLM area). A range of potential barriers to testing are currently being examined and include:

• reservations about collection of a venous sample from children

• lack of awareness or belief that lead is a health issue

• a belief that closure of the smelter will bring immediate resolution to the issue

• blood lead testing fatigue

• a desire to minimise attention and negative publicity on the NLM area.

Despite these limitations, and with a few individual exceptions, children living in closest proximity to the smelter are observed to have the highest levels of lead exposure. The use of smelter slag as landfill and wind carriage and deposition of lead emissions has resulted in various lead "hot-spots" more distant to the smelter, some of these being more than a kilometre from source. Over time the blood lead levels have improved due to a variety of factors, including efforts to control emissions by the smelter operators, and increased knowledge and awareness of residents about lead safety leading to exposure-reducing behavioural change.

An extensive range of abatement and remediation activities in homes and public spaces has been undertaken by the EHC since it began operation. These have included both a program of individual remediation of homes, aimed at identifying and removing lead from the child's home environment where children were identified with persistently high BLL, and a broad program of abatement offered first to homes close to the smelter then moving outwards. Altogether 660 homes participated in some form of abatement activity and by June 2000, 120 kilograms of contaminated dust had been removed from carpets, 9.4 tonnes of contaminated dust had been removed from ceiling cavities and 3,089 tonnes of clean soil had been used to top dress lawns and encourage grass growth.

In addition, strategies such as blood lead testing of pregnant women and children 0–13 years resident in the area, as well as promoting lead safe behaviour changes through good hygiene, nutrition and schools based programs were conducted. Further improvements in blood lead concentration are anticipated since the closure of the smelter in September 2003.

In line with growing awareness of the lead exposure issue in NLM, the smelter operator has taken various steps over the past decade to control lead dust and fugitive emissions from the smelter. They have conducted both on-site and community based monitoring of lead in air and dust deposition. Ongoing monitoring was an integral part of the company's conditions of consent to operate. Monitoring occurs daily with quarterly reporting of monthly summaries to residents, state planning, environment, and health authorities.

To some extent there appears to have been a slowing of improvement in child BLLs in the last few years since it plateaued in 1997. Whether or not this represents a general slowing in the reduction of BLLs during the last years of operation of the smelter, it is apparent that many children in the NLM area do continue to have BLLs elevated above the NHMRC defined goal of less than 10 μg/dL. This indicates a sustained need for monitoring to ensure that child blood lead levels do return to background rates following the current decommissioning of the smelter and site remediation. During this decommissioning phase, there is the potential for further contamination of the surrounding area due to a variety of factors including:

• non-routine activities including structural dismantling of the smelter

• the arrival of off-site contractors who may not be as trained or aware of lead safety and containment issues

• significant quantities of smelter slag stored on site that are still to undergo remediation.

It is possible that other sources of lead are influencing the observed findings presented here. Across Australia, child BLLs have been falling in unison with the reduction in use of lead in petrol. Ageing housing stock presents a continuing challenge in the form of peeling lead based paint and this is the major source of lead exposure across the broader community. However, a study comparing high-precision lead isotopic ratios in deciduous teeth of children living in the NLM area with environmental sources, found that in most children only a small contribution to tooth lead can be attributed to petrol and paint sources, and that between 55 to100% of lead could be derived from the smelter [13].

Within Lake Macquarie council area, the previous use of smelter slag as fill in numerous locations, both public and private, may also be contributing to elevated BLLs in some individuals. Current advice continues to be that a barrier, such as returfing and top dressing to promote grass growth, and the use of ground covers such as pine bark, be used to deny children access to slag and lead dust.

A limitation of this study is that the only available soil lead data was collected in 1992. The residential soil lead concentrations used in the analysis are those collected from this single survey, hence the analysis does not include changes to soil lead concentrations that occurred in the intervening years. Subsequent to the soil survey residential soil lead may have increased from smelter emissions or decreased from remediation. To determine the rate of surface soil lead recontamination after remediation, five soil plots were established at varying distances, up to 1 km north, south and west of the smelter. Each plot was 16 m^2^, 300 mm deep and was filled with the same coarse sandy loam used in the lead abatement program before being covered with grass turf. Annual testing prior to closure of the smelter found an average increase in lead of approximately 2 ppm per month at 0 – 25 mm depth. The average increase of 2 ppm per month (24 ppm per year) would have little impact on increasing soil lead values in the years since the soil survey in 1992.

The mean total lead in soil at 25 mm depth for the soil plots on establishment was 5.5 ppm. So there is a potentially large decrease in soil lead values after abatement that would impact on the lead exposure to children in the study, and may bias the results of this study. The complete list of properties that underwent abatement activity since the 1992 soil survey was not available for analysis. Yet the sample year analysed and used in the modelling would in effect capture some of the decreases in BLL due to abatement activity. When this data becomes available it is proposed to re-analyse the residential soil lead data and modelling with these figures.

Also, the soil lead spatial sampling frequency of 200 metres required interpolation between samples to allocate soil lead concentrations for children's residences. Ideally the soil survey sampling would have been at the location of each child residence for more accurate soil lead analysis. This factor together with residential abatement and behavioural characteristics of individual children not available for analysis, such as how often the child plays outdoors, how often hands are washed, the percentage of home-grown vegetable consumption, education level of primary caregiver, and lead-in-air levels would likely account for much of the remaining variation [6,8,9] not explained by the models presented in this paper and are a possible source of bias.

In summary, this paper illustrates the impact of lead exposure from a point source on children living in the environs. Gradients of exposure are apparent over space and time with falling lead levels indicating improvement, both in lead emissions and exposure prevention. Recent closure of the smelter source should lead to further significant reductions in lead in the surrounding environment and lower blood lead levels in child residents in the area. Whilst these data do not allow accurate ascertainment of lead levels across the total target population, they do indicate residual impact of lead exposure on many children living in the area, despite abatement activities by the EHC. Monitoring will continue to gauge whether closure of the smelter leads to further improvements, and whether it is necessary to investigate other potential sources of lead exposure such as ageing house stock or lead slag used extensively for land fill.

## Methods

Methods of both soil and blood lead testing have been described previously [2] and are summarised here. A house to house survey was used to identify children in the target area. Local schools were approached and further efforts made to engage the community in the lead testing program. Venous blood was collected and BLLs, measured as μg/dL of blood, were determined via atomic absorption spectroscopy. All children tested were entered into annual spreadsheets listing the child's name, age, sex, address and BLL result. All children residing in the area were invited to attend annually for BLL testing and where a child was identified with an elevated BLL (greater than10 μg/dL) various interventions were initiated and follow-up testing arranged to monitor the child closely. However, where children had multiple tests in a one year period only the highest BLL was reported.

Child residential address data was geocoded using MapInfo's MapMarker [14] software (geocoding is the process of converting a street address to geographic coordinates, which are then used to locate the address on a map). The geocoded data was then imported into MapInfo Professional (MiP) [15] and various imaging and point presentations of the data created. The linear distance of each residential address from the smelter complex was calculated using standard MiP functionality. Thematic maps of child BLLs were created using MiP for visual assessment and presentation.

The level of lead in soil surrounding the smelter was assessed in 1992 via collection of 202 soil samples from intersectional points of a 200 metre grid covering an area approximately 2 km north, south and east of the lead smelter [3] (see Figure 2). No population centres exist immediately to the west of the smelter site, and land available for development is limited to the southeast by an escarpment – Munibung Hill. Soil samples were analysed by three laboratories using nitric acid digest and atomic absorption spectroscopy techniques.

Soil lead levels were collected for each intersectional point of the soil grid, measured in parts per million (ppm) of lead per total soil. The sample locations were digitised and linked to a geographic marker of the actual survey grid. Grid point coordinates were then calculated programmatically and data imported into ERMapper v6.4 [16] for grid processing and analysis. The soil cut-off limits shown in the image display in Figure 2 were chosen to enhance the visual representation of the soil survey data, and are different from the limits chosen for categorical data analysis. However, soil cut-off limits for both image display and categorical data analysis include the National Environmental Protection Council's Health Investigation Level of 300 ppm and the former NHMRC acceptable level of 1000 ppm soil lead. The ERMapper grids were then imported into MiP for mapping and comparison with blood lead levels (BLLs). The soil lead concentration for each residential address was interpolated from the soil lead grid using Encom Technology's MapEase 2.0 [17] add-on for MiP.

The residential distance from smelter and residential soil lead data was imported into SAS v8.2 [12] software for analysis. Residential soil lead data was categorised into the following ranges:

0 – < 150 ppm

150 ppm – < 300 ppm

300 ppm – < 1000 ppm

1000 ppm – < 2000 ppm

≥ 2000 ppm

Residence outside soil survey area

Residential distance from the smelter was categorised into the following ranges:

0 km – < 1 km

1 km – < 1.5 km

1.5 km – < 2.0 km

2.0 km – < 2.5 km

≥ 2.5 km

Eight children 13 years or older were not included in the analysis. Simple univariate measures for elevated BLL were calculated against four indicators; residential distance from smelter, residential soil lead concentration, year of BLL sample and age group. Preliminary assessment of BLLs showed a log-normal distribution, hence geometric means plus 95% confidence intervals were calculated for both the total sample and for specific indicators.

Linear regression models were fit for BLL using the REG procedure in SAS v8.2 [12]. A non-linear relationship between BLL and residential soil lead was observed so the log of residential soil lead concentration was used in the model. It was also observed in Figure 9 that there was a non-linear relationship between BLL and age, with BLL increasing from birth until 3 years of age where it then decreased, so higher order polynomial terms of age (quadratic and cubic) were included in the model. The final model was fit for BLL using the explanatory variables; residential distance from smelter, year of BLL sample, age, higher order polynomial terms of age, and natural log of residential soil lead concentration.

## Competing interests

The author(s) declare that they have no competing interests.

## Authors' contributions

AW geocoded residential addresses, digitised the soil lead survey data, calculated grids of soil lead concentration, interpolated values of soil lead concentration of residence for each child sampled, calculated residential distance from the smelter, calculated univariate statistics of BLL against all indicators, generated models of BLL, and assisted drafting the manuscript.

TS drafted the manuscript, obtained, cleaned and summarised BLL data from the testing laboratory, and assisted with geocoding and analyses.

LB provided information and commentary on the abatement, lead safety actions and blood lead testing in the NLM and assisted drafting the manuscript.

CD provided advice on sampling and analysis, and assisted drafting the manuscript.
